# Barriers Implementing Patient‐Specific 3D‐Printed Bone Scaffolds in Australia: A Scoping Review

**DOI:** 10.1155/ijbm/5250806

**Published:** 2026-04-06

**Authors:** Anthony Vidler, Angus Hayes

**Affiliations:** ^1^ School of Rural Medicine, Charles Sturt University, Orange, New South Wales, Australia, csu.edu.au; ^2^ Sunshine Coast Health Service, Queensland Health, Birtinya, Queensland, Australia, health.qld.gov.au; ^3^ New South Wales Health, Sydney, New South Wales, Australia

**Keywords:** additive manufacturing, bioprinting, implantable devices, orthopaedics, three-dimensional, tissue scaffolds

## Abstract

3D printing has emerged as an innovative technology over the last decade, with widespread uptake in several fields spanning science, technology and engineering; however, penetration into medical markets has been met with a variety of obstacles. At the 17^th^ Global Conference on Sustainable Manufacturing in 2018, medical technology stakeholders held a workshop to discuss the barriers that hinder the widespread adoption and diffusion of 3D printing technology in the Australian medical field. Patient‐specific 3D‐printed bone scaffolds were the central focus of this workshop, with five major barriers identified: material issues; manufacturing and postprocess approval; medical and professional endorsement and adoption; reimbursement; and staff training. To determine the progress made towards overcoming these barriers, we have reviewed the body of literature published from the 2018 conference until now. The most significant progress was observed in material issues with a significant increase in the number of materials and combination of materials that have been successfully printed and used in multiple animal trials and limited human cases. Manufacturing and postprocess approval issues that enable 3D‐printed bone scaffold implantation have seen preliminary success with a handful of case trials documented with various levels of success, yet a distinct level of development is still required to satisfy this barrier before commercial production parameters and widespread adoption will be recognized. Medical and professional endorsement and adoption, reimbursement and staff training are yet to experience significant progression, with the possibility that the extent of these barriers will not be clearly understood until well after regulatory approval has been achieved.

## 1. Introduction

Patient‐specific 3D‐printed bone scaffolds (3DPBS) are customized structures designed using 3D printing (3DP) technology that mimics the anatomical and structural characteristics of an individual patient’s anatomy to treat a bone defect or injury. These scaffolds serve as foundational frameworks, promoting bone regeneration and providing personalized solutions for improved patient outcomes [1–4].

3DPBS are increasingly proposed as a transformative technology in regenerative medicine as well as reconstructive and orthopaedic surgery. Experts in the field acknowledge their potential to overcome the limitations of traditional bone grafting techniques by enabling the creation of patient‐specific implants that precisely match anatomical defects. This level of customization proposes improved surgical accuracy, enhanced integration with native bone and faster patient recovery.

Financially, the technology has the potential to generate cost savings for the Australian healthcare system. If reduced complications, shorter hospital stays and decreased rates of revision surgeries are realized from implementing 3DPBS, overall expenditure stands to fall despite what may prove to be a higher upfront cost. Furthermore, local development and production of 3DPBS could support the growth of the biomedical manufacturing sector, contributing to Australia’s innovation economy and reducing reliance on imported medical devices.

3DPBS integration trials have already shown positive outcomes through in vitro studies, animal studies and a small number of human trials [1–4]. However, despite the rapid advancement of the technology, multiple barriers still exist that impede the widespread implementation of 3DPBS in Australia.

The 17^th^ Global Conference on Sustainable Manufacturing brought together stakeholders within the medical technology industry to discuss the additive manufacturing of medical devices in Australia. Among numerous goals, two aims were to grow the scale of the medical technology industry through additive manufacturing and offer Australians potential treatment options derived from 3DP. 3DPBS was, as a test case, used to identify unique barriers to the Australian healthcare landscape that must be overcome before 3DP would be widely adopted as a treatment option [5].

Of the barriers discussed, stakeholders identified five as being the most pertinent in restricting the advancement and adoption of 3DPBS.

Material issues (MI), staff training, manufacturing and postprocess approval (MPPA), medical and professional endorsement and adoption, and medical device reimbursements were widely agreed upon as being the greatest barriers for the widespread adoption of 3DPBS [5].

Since 2018, the body of work surrounding additive manufacturing of orthopaedic implants has grown exponentially, and in turn, so has the expectation that patient‐specific 3DPBS will translate into common clinical practice. To our knowledge, an assessment of the progress towards overcoming the identified barriers is yet to be made. The aim of this review was to amalgamate the most recent literature in the field of 3DPBS, appreciate the advancements that have taken place and draw focus to future work required before 3DPBS can become a widely accessible treatment option for the Australian population.

This scoping review takes a narrow focus on the Australian health care only, excluding the consideration of international healthcare systems, which was seen to be warranted because the adoption of additive manufacturing in Australian health care is shaped by Australia’s distinct regulatory framework (TGA), market scale, local manufacturing industry structures and reimbursement pathways. An international focus would introduce heterogeneous regulatory, economic and health system contexts; diluting relevance; obscuring Australia‐specific barriers; and limiting the applicability of findings to local policy, industry and clinical translation.

## 2. Methodology

### 2.1. Search Strategy

**FIGURE 1:**
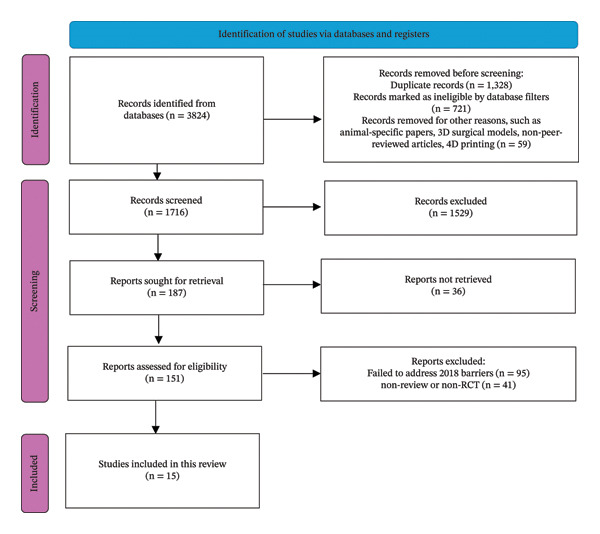
Process of literature exclusion (PRISMA).

The search strategy was conducted in accordance with the Joanna Briggs Institute (JBI) Scoping Review Guidelines [6]. A comprehensive search of electronic databases by the author (AV) was conducted throughout February 2024. Medline (Ovid) and PubMed were searched using MeSH terms and Boolean operators. Cochrane Database of Systematic Reviews and Google Scholar were searched with keywords. The scope of research was limited to studies with a publication date of 2018 or later. Additional studies were identified via hand‐searching methods, such as journal screening as well as reference list reviews and citations tracking of prominent articles (Figure 1). All studies were exported to EndNote, and duplicates were removed. Results were recorded and reported via the PRISMA [7] framework as endorsed by JBI [6]. PRISMA guidelines were employed to ensure evidence‐based reporting standards, ensuring transparency, completeness and reproducibility of this scoping review.

#### 2.1.1. Medline Search Strategy

1. exp Bioprinting/or exp Printing, Three‐Dimensional/13994
2. exp Tissue Scaffolds/or bone scaffold.mp. or exp Bone Substitutes/39468
3. 1 and 2 3197

### 2.2. Study Selection

All titles and abstracts of the identified studies were independently screened by the author (AV) and co‐author (AH) against the following criteria, with discrepancies between the two assessors included for full‐text screening (Figure 1):

1. Published in English;
2. Systematic reviews, scoping reviews, narrative reviews, meta‐analysis and randomized controlled trials;
3. Studies specifically assessing 3DPBS.

All abstracts that met the above criteria were full‐text screened by author and co‐author independently, and studies that failed to unanimously meet the exclusion criteria below were discussed in detail and either included or omitted accordingly (Figure 1):

1. Full text was unavailable via open access through the Council of Australian University Librarians (CAUL) or Google Scholar.
2. Studies did not specifically investigate MI, staff training, MPPA, medical and professional endorsement and adoption, and medical device reimbursements of 3DPBS (Figure 1).

### 2.3. Ethics

Ethics approval was not required for this review.

## 3. Results

Search results yielded 15 studies that were deemed suitable for inclusion in this review (Table 1). Fourteen of the 15 articles propose solutions for overcoming the MI barrier, while 6 of the 15 articles present solutions to the various characteristics of the barrier presented by MPPA. No articles were identified that addressed the barriers of staff training, medical and professional endorsement and adoption, and medical device reimbursement; therefore, no findings are presented in this section.

**TABLE 1:** Articles included for review.

Author	Article type	Year	Country	Findings
Zhang et al. [3]	Literature review	2021	International	MI—identify the advantages and disadvantages of metallics, bioceramics and composite materials and their associated printing methods.
Shiva et al. [8]	Literature review	2023	International	MI—identify the advantages and disadvantages of printing with polymers and current medical applications of the material.
Xu et al. [9]	Literature review	2022	China	MI—identify the advantages and disadvantages of polymers, metallics, bioceramics and composite materials and their associated printing methods
Garot et al. [10]	Literature review	2021	USA	MI—identify the advantages and disadvantages of polymers, metallics, bioceramics and composite materials and their associated printing methods MI/MPPA—cites human trials of various 3DPBS MPPA—cites approvals by the USA Food and Drug Administration. MPPA—cites proven sterilization methods relevant to surface finish component of manufacturing and postprocess approval barrier
Zhang et al. [11]	Mini‐review	2019	USA	MI—assess material‐specific advantages and limitations of metallics and bioceramics, including associated printing technologies.
Kanwar and Vijayavenkataraman [12]	Literature review	2021	USA/UAE	MI—analyse the strengths and weaknesses of metallic and bioceramic materials for proposed 3DPBS and their corresponding printing approaches. MPPA—discusses methods for 3DPBS surface optimization
Bahraminasab [13]	Literature review	2020	Iran	MI—assess the pros and cons of specific printing methods that exist when using materials that are commonly proposed for 3DPBS.
Ma et al. [14]	Literature review	2018	China	MI—outline the benefits and limitations of materials in conjunction with their printing methods with a particular focus on bioceramic and composite materials.
Qu et al. [15]	Literature review	2021	USA/China	MI—focus on evaluating the benefits and drawbacks of metallic materials and the advantages and disadvantages related to multiple printing methods. MPPA—discusses methods for 3DPBS surface optimization
Chae and Cho [16]	Literature review	2023	South Korea	MI—investigate the suitability of polymers as a suitable material for 3DPBS and the associated limitations of printing methods currently available
Shim [17]	Literature review	2023	South Korea	MI—compare currently proposed materials for 3DPBS highlighting their strengths, weaknesses and the potential limitations of printing methods in a commercial sense.
Ivanovski et al. [18]	Literature review	2023	Australia	MI—investigate the suitability of titanium 3DPBS and the challenges in printing titanium scaffolds. MI/MPPA—cites human trials of various 3DPBS
Mirkhalaf et al. [19]	Literature review	2023	Australia	MI—review how material composition and printing methods influence scaffold properties and highlight key translational challenges
Freeman et al. [20]	Literature review	2021	Ireland	MI—investigate the suitability of ceramics and polymers for use in 3DPBS. MPPA—discusses methods for 3DPBS surface optimization
Chakraborty [21]	Literature review	2023	India	MPPA—discusses methods for 3DPBS surface optimization

Abbreviations: MI, relevant to material issue barrier; MPPA, relevant to manufacturing and postprocess approval barrier.

MI focus on identifying optimal combinations of materials and printing techniques to create bone scaffolds that closely replicate the mechanical and biological characteristics of natural bone. Furthermore, these challenges involve evaluating the availability and cost‐effectiveness of these material and printing combinations for large‐scale production [5]. Our literature search identified several materials and printing combinations that are currently considered acceptable for 3DPBS (Table 2).

**TABLE 2:** Material/printing method combinations suitable for 3DPBS and their relevant challenges.

Material suitable for 3D‐printed bone scaffolds	Suitable printing methods	Elements of concern
*Polymers*		
PEEK [8–10]	SLS [8, 9, 11, 12] FDM [8–10, 13, 14] SLM [15]	Nonbiodegradable [8] High cost [3, 8, 10, 11, 14, 16, 17] Slow printing speed [3, 8, 11] Brittle [8, 10] Imaging interference [8]

*Metallics*		
Titanium [3, 10–12, 15, 17–19]	SLS [3, 10–12, 17, 19] EBM [3, 10–12, 17, 19]	Slow and complicated printing process [3, 17] High printing cost [3, 14, 17] Delayed osteogenesis [3, 19] Nonbiodegradable [3, 10, 12, 19]
Tantalum [3, 11, 15, 17, 19]	SLS [3, 10–12, 17, 19] EBM [3, 11, 17, 19]	Slow and complicated printing process [3, 17] High printing cost [3, 17] Delayed osteogenesis [3, 19] Nonbiodegradable [3, 12, 19]

*Bioceramics*		
TCP [3, 10–12, 17, 19]	SLS [3, 13, 14, 19] FDM [17, 19]	Brittle [3, 10–12, 14, 19] High cost [3, 11, 14, 16, 17] Slow degradation [3, 10, 11] Slow printing speed [11, 16]
HAP [3, 10–12, 14, 17]	SLS [3, 13, 14] SLA [10]	Brittle [3, 10, 12, 14, 19] Slow degradation [3, 10] Slow printing speed [11]

*Composite materials*		
PLA/HAP [3, 10, 14, 17, 20]	SLS [3, 10, 14, 15]	High cost [3, 14, 16, 17] Technically challenging to produce [3, 16] Slow printing process [3, 16]
TCP/PCL [10, 12, 14, 21]	SLS [3, 10, 11, 14, 15]	High cost [3, 14, 16, 17] Technically challenging to produce [3, 16] Slow printing process [3, 16]

*Note:* SLA, stereolithography.

Abbreviations: EBM, electron beam melting; FDM, fused deposition modelling; SLM, selective laser melting; SLS, selective laser sintering.

MPPA as a barrier refers to the key hurdles to the adoption of 3DPBS in Australia. In 2018, additive manufacturing experts identified critical factors, such as quality control, repeatability, surface finish and validation. Quality control ensures implants meet safety standards, while repeatability guarantees precise production. Surface finish includes optimization (debulking, porosity and biological treatment) and sterilization to ensure implants are safe and intact. Validation requires approval from regulatory bodies, such as the FDA (USA) or TGA (Australia) [5]. Our search identified multiple articles that offer solutions to components of this barrier (Table 3).

**TABLE 3:** Fulfilment of quality control parameters for 3DPBS.

Material	Quality control	Repeatability	Surface finish		Validation
			Sterilization	Optimization	
PEEK	Yes [10, 18]	—	(*γ*) Irradiation [10]	—	FDA^†^ [10]
Titanium	Yes [10, 18]	—	Autoclaving [10]	—	—
Tantalum	Yes [10, 18]	—	Autoclaving [10]	—	—
TCP	Yes [10, 18]	—	(*γ*) Irradiation [10]	—	—
HAP	Yes [10, 18]	—	(*γ*) Irradiation [10] Ethylene oxide [10]	—	—
PLA/HAP	—	—	(*γ*) Irradiation [10]	—	—
PCL/TCP	Yes [10, 18]	—	(*γ*) Irradiation [10] Ethylene oxide [10]	—	—

^†^Approval for craniomaxillofacial applications only.

## 4. Discussion

### 4.1. MI

MI revolve around the identification of effective combinations of materials and printing modality to produce a bone scaffold with mechanical and biological properties that most closely represents the native bone it replaces. Additionally, MI are concerned with the potential availability and cost‐effectiveness of material and printing mode combinations if production were to be upscaled [5].

Successful bone scaffolds require the product of the material/s and printing method to mirror the mechanical properties of bone, including elasticity, shear strength, compressive strength and tensile strength [12, 13, 19]. Further, they must support the biological functions of human bone, such as osteoblastic and osteoclastic activity, vascularization and calcium phosphate balance [11, 12, 14, 16]. In addition to achieving this delicate balance, materials must also produce a scaffold that is anatomically precise, free from toxicity and that does not produce an inflammatory response. Given the diverse mechanical and biological requirements of various bones in the body, versatility in materials is essential to accurately replicate each bone’s properties [12, 16].

Various combinations of materials and printing modalities have been shown to possess the characteristics and features that make them suitable for use as a 3DPBS, yet each of these presents challenges to overcome. Polymer materials are a common material used in 3DP. A polymer material is a large molecule composed of repeating structural units called monomers, which are bonded together to form long chains, giving polymers their unique properties, such as flexibility, durability and versatility. For 3DPBS, polyether ether ketone (PEEK) is widely accepted as a suitable polymer [8–10], and scaffolds made of PEEK have undergone multiple clinical trials [10]. Additionally, PEEK can be printed via multiple methods, enhancing adaptability to different applications and facilities [8, 10–14]. However, PEEK scaffolds have been observed to be brittle, create interference when imaged and have poor biodegradability [8, 10]. Furthermore, high printing costs and slow printing speeds hamper the clinical suitability of PEEK bone scaffolds [3, 8, 10, 11, 16, 17].

Metallic materials have a proven clinical application as bone scaffolds, in which titanium and tantalum are widely reported and agreed upon for their ability to act as a suitable material for bone scaffold production [3, 10, 12, 15, 17–19]. Despite their widespread adoption, multiple issues still exist when considering the use of titanium and tantalum as scaffold materials in isolation. Both materials are poor at inducing osteogenesis, which delays the healing process, and neither is biodegradable, meaning consecutive surgeries may be required if the scaffold is only temporary [3, 10, 12, 19]. Furthermore, the process for printing titanium and tantalum is protracted, complicated and costly [3, 14, 17].

Bioceramics are defined as ceramic materials that are compatible with biological systems. This material class bears much promise as a bone scaffold medium. Ceramic materials are neither metallic nor organic, often displaying crystalline, glassy or a combination of both structures. They are generally hard and chemically inert, capable of being shaped or consolidated through heating processes [22].

Bioceramics that have been proven to be an acceptable substrate for bone scaffolds—supported by successful human case studies—are tricalcium phosphate (TCP) and hydroxyapatite (HAP) [10, 18]. However, despite clinical evidence, TCP and HAP both exhibit significant disadvantages. Bone scaffolds manufactured from 100% TCP or HAP have been shown to be brittle and degrade at a slower rate than optimal. Both TCP and HAP are slow to manufacture, with TCP also being expensive [3, 10–12, 14, 16, 17, 19].

Beyond the manufacture of bone scaffolds from individual materials, progress has been made in developing scaffolds from composite materials. Multiple iterations of various compositions are currently being investigated; however, polylactic acid (PLA) combined with HAP [3, 10, 14, 17, 20], and TCP combined with polycaprolactone (PCL) [10, 12, 14, 21] are two emerging combinations that show promise. However, despite enhancements made by combining materials, the introduction of multiple materials into a single scaffold does little to resolve the production challenges, nor does it reduce costs or improve time efficiency [3, 14, 16, 17].

MI are the domain that has seen the greatest development. This comes as no surprise, as identification of material/s and printing combination that produces bone scaffolds that are biomimetic of native bone is the logical first step in the development process. Through our review of the literature, the authors identified several material and printing combinations that are widely accepted as being fit for purpose. Although our list of findings is not exhaustive, it does provide a detailed picture of the current status of whether the barrier of “materials issues” has been overcome. 3DPBS use in human trials, when considered concurrently with limited regulatory approvals, suggests that this barrier is being slowly overcome. However, it is apparent from the literature that the current suite of materials and printing methods is less than optimal. Currently, all proposed materials and printing combinations for bone scaffold production require a compromise to be made by both medical teams and patients, suggesting that while 3DPBS are indeed viable, they translate poorly to the clinical setting in their current form. Further development of materials is required before the 3DPBS are a first‐line option in Australia, or even globally. The current literature suggests that the mechanical properties of bone scaffolds can be overcome, and for nonbiodegradable implants, such as PEEK, and titanium and tantalum scaffolds, this is where an engineering advantage exists. However, biodegradable scaffolds require structural and biological properties, and many variables are yet to be completely understood and accommodated [12, 14, 16, 20]. While composite material selection poses a significant challenge to 3DPBS uptake, production further amplifies these issues. Recent advancements in artificial intelligence (AI) and machine learning (ML) may hold the key to accelerated progress in the MI domain. Recent studies suggest that the application of AI and ML can be employed to accurately simulate biological environments, optimize material composition and monitor implant performance in real time. This suggests that use of AI and ML can help drive material designs, particularly in the case of composite materials where titrating quantities of individual materials lead to variances in biomechanical performance. Through performance simulation before production and implantation, AI and ML can not only reduce the time and financial expenditure in the development phase of the technology but also hold the ability to further customize 3DPBS when products eventually reach the market [23].

In 2018, one of the variables that contributed to MI as a barrier was the influence of increased production scales on the cost of raw materials and printers [5]. While a number of papers commented on the cost of production, none were based on forecasted production levels when 3DPBS become clinically available and demand for the intervention increases. This suggests that despite a forthcoming proof of concept, clinical translatability still requires consideration of logistical parameters and unique solutions for the Australian healthcare landscape.

### 4.2. MPPA

MPPA addresses variables that pose a significant barrier to the adoption of 3DPBS in Australia. In 2018, professionals in the additive manufacturing space identified the variables of quality control, repeatability, surface finish and validation as most important in overcoming this barrier [5]. Quality control refers to the production of an implant that is acceptable to be implanted to a human patient. Repeatability refers to the ability of an implant to be produced precisely to its mechanical, structural and biological blueprint. Surface finish details the establishment of a definitive process that a scaffold undergoes postprinting in preparation for implantation. To assess this, surface finish is divided into two subcategories: optimization and sterilization. Optimization is achieved through debulking, porosity specifics and biological factor treatment, while sterilization identifies an effective method for eliminating viable microorganisms from the scaffold without compromising the integrity of the implant. Finally, validation refers to the established approval by regulatory authorities, such as the Food and Drug Administration (USA) or Therapeutic Goods Administration (Australia) [5].

#### 4.2.1. Quality Control

Of the material/printing combinations described earlier, PEEK, titanium, tantalum, TCP, HAP and PCL/TCP have displayed a level of quality that provides surgeons with confidence where they feel the use of these scaffolds is acceptable, evidenced by various case studies, such as PEEK scapular implant in a 16‐year‐old boy with benign, fibrous histiocytoma, a study of six patients receiving 3DP vertebrae and an 11‐year‐old patient on receiving a PCL/TCP implant for a large cranial defect [10, 18]. Quality control, however, does not imply repeatability. Quality control merely implies that a single scaffold has been assessed to satisfactorily possess an adequate level and combination of mechanical, structural and biological properties to replace and perform like the native bone of the recipient. For clinical translation, quality control and repeatability must exist in tandem with a level of autonomy and confidence that reduces manufacturing time and increases surgical confidence. This is widely supported in the literature particularly in the aforementioned case studies where all researchers acknowledge that increased cohorts are imperative before clinical translation can be claimed a success.

#### 4.2.2. Repeatability

For widespread adoption of 3DPBS, the manufacturing process must demonstrate that the product is within acceptable limits of the design to ensure regulator, surgeon and patient confidence, and safety. Repeatability is a measure of consistent precision and accuracy of the produced scaffold relative to the anatomical and functional parameters set during the design process [24].

Attaining high levels of repeatability is further complicated for patient‐specific bone scaffolds as each scaffold is unique in its anatomy, composition and specific patient factors, suggesting that any method for assessing the precision and accuracy of 3DPBS must have the ability to consider these variables.

We were unable to establish strong evidence to suggest that any of the scaffold material or production method combinations pose an acceptable level of repeatability. Equally, we were unable to identify any guidelines for manufacturers that define acceptable variances in precision. Paucity of such evidence implies that collaboration between stakeholders is required to determine a model for assessing precision and accuracy of every scaffold produced before its implantation before the barrier of repeatability can confidently be overcome.

#### 4.2.3. Surface Finish

Surface optimization in 3DPBS is vital for enhancing biological interactions and ultimately improving implant success rates. Optimized surface characteristics, such as porosity, roughness and topographical features, can promote cell attachment, proliferation and differentiation, which is crucial for bone tissue regeneration [12, 16, 20, 21]. By mimicking the natural extracellular matrix, these surfaces encourage osteogenic activity, leading to better integration with surrounding tissues, and improved implant stability and longevity [12, 16, 21]. Methods of surface optimization have been heavily researched; however, a conclusive and universally accepted method is yet to be published for any of the scaffolds discussed in this paper. In addition to surface optimization, sterilization needs to be carried out in a manner that is effective against harmful microorganisms, without impairing the desired mechanical, structural and biological components of the scaffold. We were able to establish that (γ) irradiation (PEEK, TCP, HAP and PLA/HCP), autoclaving (titanium and tantalum) and ethylene oxide (PCL/TCP and HAP) were recognized as effective methods of scaffold sterilization [10]. However, steam, ethylene oxide and (γ) irradiation each present limitations when applied to 3DPBS. Steam sterilization, although common and cost‐effective, risks degrading heat‐sensitive materials and distorting scaffold structures. Ethylene oxide, with its lower temperatures, is more compatible with delicate polymers, but raises concerns about residual toxicity and the difficulty of fully sterilizing complex internal geometries. (γ) Irradiation offers deeper penetration and is less dependent on temperature but may weaken certain polymers through material degradation [25, 26].

Given these trade‐offs, sterilization is not merely a procedural hurdle but a fundamental barrier to safe and reliable clinical use. As material science advances, specific focus on effective sterilization methods will be needed, especially in the case of composite materials where individual materials have conflicting suitability to a process of sterilization. For manufacturers, this means tailoring an approach to sterilization will be essential to ensure sterility, preservation of function, regulatory approval and clinical trust in Australia.

#### 4.2.4. Validation

Validation refers to satisfying all other parameters for obtaining regulatory approval. Although we were unable to identify TGA approval for any 3DPBS, we found evidence that PEEK 3DP scaffolds had been granted FDA approval for limited craniomaxillofacial applications [10]. In regard to the Australian medical landscape, this holds significance as 3D‐printed implants can gain approval from the TGA through conformity assessment—an evaluation which considers a manufacturer’s demonstrated compliance with relevant standards and regulations of an international regulator, such as the FDA or EU MDR (European Medical Device Regulation) as a positive contributor towards approval. This typically involves providing evidence of the implant’s safety, quality and performance through clinical data, risk assessments and manufacturing processes [27].

Despite existing case studies, it should not be assumed that all variables of materials, manufacture and postprocessing are satisfied or that full regulatory approval has been granted. Such case studies gain limited approval based on expanded access avenues. Expanded access allows patients with serious conditions to access investigational medical devices that are not yet approved, offering potential treatment options outside of clinical trials under certain circumstances, typically with regulatory oversight [28, 29].

### 4.3. Medical and Professional Endorsement and Adoption

Professional endorsement and adoption were seen to be a significant barrier to the diffusion of 3DPBS in Australia [5]. Many factors play a role in the adoption of a new technology in hospitals; however, the 2018 conference identified that a surgeon’s endorsement is the most influential factor in the decision‐making process when adopting a new technology within surgical departments [5, 29]. While many studies exist outlining the factors that create barriers to professional adoption of new medical technology, our search failed to identify any reliable studies that assessed the willingness of medical professionals to implement 3DPBS in their current form into clinical practice.

It should be noted that through our search, we identified a recent study, titled “A Clinical Risk Assessment of a 3D‐Printed Patient‐Specific Scaffold by Failure Modes and Effects Analysis,” that concluded medical professionals were either satisfied or neutral regarding the current state of titanium 3DPBS for alveolar reconstruction. However, of the 41 participants surveyed, only 13 were surgeons directly responsible for the implantation of such devices with results not reporting the satisfaction rating of surgeons as a subset within the cohort, therefore limiting the conclusiveness of the findings relative to our study [30].

The general paucity of findings is not unexpected, as diffusion and adoption of new technologies relies on an approved device to be present in the market before widespread product roll‐out can be initiated. For medical professionals, this will rely on overcoming the yet‐to‐be‐satisfied barriers discussed in Sections 4.1, 4.2, 4.4 and 4.5. The timing and approach for gaining endorsement from Australian surgeons will depend on each 3DPBS developer’s goals and market entry plans. However, given Australia’s relatively small market size and limited pool of specialist expertise, developers who proactively engage and secure support from influential local surgeons are likely to have a competitive edge and be more successful in gaining market share [31].

### 4.4. Medical Device Reimbursements

Medical device reimbursement as a barrier is defined as the financial incentive to applying 3DPBS as a treatment. In Australia, Medicare (Australia’s universal health insurance scheme) and often private health fund payments are only available to devices that have the current approval of the TGA [32]. As we were unable to identify any 3DPBS that hold current TGA approval, a financial incentive beyond research funding is nonexistent in the Australian healthcare system. Our search failed to locate any literature that identifies current financial incentives for practitioners to implement 3DPBS in their current form. Regardless, some progress has been made towards streamlining the approval process for 3DP implantable devices since the 2018 conference. Forward from 2018, consultations with industry stakeholders have taken place, and the TGA has updated their regulatory definitions and processes for 3DP implantable personal devices, with the view to fairly accommodate what would appear to be impending future approval requests of 3DPBS. While this does not signify that the barrier of medical device reimbursements has been overcome, it paves the way for reduced resistance in the future [33]. Beyond regulatory approval, it is yet to be seen if current government or private reimbursement policies can accommodate such highly individual surgical therapies when a tenable product is presented to the Australian market. Therefore, to support the safe and effective integration of 3DPBS and similar innovations into mainstream care, reimbursement models must evolve alongside regulatory progress. A collaborative approach involving regulators, insurers, clinicians and industry stakeholders is essential to ensure that funding structures are responsive to technological advancements and capable of delivering long‐term value to patients and the healthcare system.

### 4.5. Staff Training

Staff training as a barrier is the deficit in human resources that impedes the adoption and diffusion of 3DPBS. A lack of defined roles and responsibilities across all domains of imaging, engineering, surgical intervention and recovery exists in the current 3DPBS landscape. We identified multiple articles that explore a vast number of potential variables detailing staff training as a barrier. However, these articles fail to identify a resolution for any of these variables. The scarcity of literature regarding resolution is unsurprising, as while potential human resource challenges can be hypothesized, the true deficits of qualified staff cannot be realized until clinical translation of 3DPBS becomes a reality, and increased levels of production have begun. Despite the lack of understanding of what specific human resources will be required to effectively deliver 3DPBS, the common manpower challenges that broadly exist in the Australian healthcare system today will inevitably impact how 3DPBS are adopted. It has been documented that in Australia, up to 80% of health profession occupations face a worker shortage [34] which in turn contributes to staff attrition and a reduced capacity for additional training [35]. While we acknowledge that these workforce trends do not require solving by 3DPBS manufacturers, we insist that manufacturers who consider such factors when designing the delivery of their products will be more successful.

## 5. Future Research Direction

Clearly, future research direction lies in the optimization of material and printing combinations to develop bone scaffolds that are both mechanically robust and biologically functional, while remaining cost‐effective and clinically translatable at scale. Current materials, such as PEEK, titanium, tantalum, TCP and HAP, each present significant limitations, particularly around biodegradability, brittleness, osteogenesis and production costs, highlighting the ongoing challenge of MI. Composite materials offer promise but still face issues of efficiency, repeatability and scalability, underscoring the importance of advancing MPPA. Future work must therefore focus on creating versatile, biomimetic composites with reliable manufacturing processes, while simultaneously addressing sterilization, regulatory approval pathways and health‐economic modelling to support both medical device reimbursement and clinical translation.

While future research must concentrate on resolving the scientific and technical barriers of MI and MPPA, parallel progress in policy and funding support will be critical. Scientific innovation alone cannot guarantee successful adoption without structural mechanisms to reduce financial risk, enable clinical validation and prepare the healthcare workforce for issues that directly relate to staff training and the need for medical and professional endorsement and adoption.

One solution to this may lie in the Australian Government’s Medical Science Co‐Investment Plan, particularly at this stage of 3DPBS development. In essence, the co‐investment scheme aims to reduce financial pressure during the high risk, pre‐reimbursement phase, accelerating evidence generation and providing a platform for validating and scaling innovations, such as 3DPBS. This may prove successful in making it easier for companies, especially international medical device firms, to overcome reimbursement hurdles and bring proposed implants into mainstream clinical practice [36]. The Australian Government’s co‐investment scheme supports not only the development of technologies, such as 3DPBS, but also their future adoption in clinical practice. By funding training pipelines and fostering collaboration with universities and hospitals, the scheme may help build a 3DPBS skill‐specific workforce of surgeons, engineers and technicians familiar with scaffold design and application, addressing the staff training barrier. Engagement with peak bodies and clinicians during development encourages early professional endorsement, while government backing lends credibility and reduces barriers to hospital uptake. Funding also enables clinical trials, pilot programmes and training modules that generate real‐world evidence and prepare clinicians before regulatory approval. Together, these measures suggest that once approved, 3DPBS producers are supported by staff expertise, medical consensus and a strong ecosystem for adoption, partially addressing all five major barriers to 3DPBS integration.

## 6. Limitations

This review is limited by the relative infancy of additive manufacturing for implantable devices. It was evident from reviewing current literature that standardized terminology is yet to be established, suggesting that some relevant articles may not have been discovered during our extensive search. Additionally, much of the literature currently relates to material and manufacturing properties that are universal the world over. However, when assessing progress on the barriers that possess a geographical element, such as staff training, MPPA, medical and professional endorsement and adoption, and medical device reimbursements, assessments can at best be hypothetical while any product is yet to be presented to the Australian market.

## 7. Conclusion

Since the 17^th^ Global Conference on Sustainable Manufacturing in 2018, many developments have occurred towards the adoption and diffusion of 3DPBS. However, despite these advancements, widespread clinical translation of the technology is still years away. Vast developments surrounding MI have been made but at this stage are far from optimal. Inherently, this inhibits progress required in the fields of MPPA, medical and professional endorsement and adoption, medical device reimbursements and staff training. For 3DPBS to be a widely accepted method of treatment, MI require suitable resolution so that advancement in subsequent fields can be initiated, and a reliable and financially viable patient‐specific 3DPBS can be presented to the Australian healthcare market.

**Nomenclature**
3DPBS: Patient‐specific 3D‐printed bone scaffolds
3DP: 3D printing
JBI: Joanna Briggs Institute
PRISMA: Preferred Reporting Items for Systematic Reviews and Meta‐Analyses
FDA: Food and Drug Administration of America
TGA: Therapeutic Goods Administration of Australia
PEEK: Polyether ether ketone
TCP: Tricalcium phosphate
HAP: Hydroxyapatite
PLA: Polylactic acid
PCL: Polycaprolactone
EU MDR: European Medical Device Regulation
SLS: Selective laser sintering
FDM: Fused deposition modelling
SLM: Selective laser melting
EBM: Electron beam melting
SLA: Stereolithography
AI: Artificial intelligence
ML: Machine learning

## Author Contributions

Conceptualization: Anthony Vidler.

Methodology: Anthony Vidler and Angus Hayes.

Validation: Anthony Vidler and Angus Hayes.

Formal analysis: Anthony Vidler and Angus Hayes.

Investigation: Anthony Vidler.

Resources: Anthony Vidler and Angus Hayes.

Data curation: Anthony Vidler and Angus Hayes.

Writing–original draft preparation: Anthony Vidler.

Writing–review and editing: Anthony Vidler.

Visualization: Anthony Vidler.

## Funding

No funding was received for this research. Open access publishing was facilitated by Charles Sturt University, as part of the Wiley–Charles Sturt University agreement via the Council of Australasian University Librarians.

## Disclosure

All authors have read and agreed to the published version of the manuscript.

## Ethics Statement

An ethical approval was not required for this review.

## Conflicts of Interest

The authors declare no conflicts of interest.

## Data Availability

Data sharing is not applicable as no new data are generated.
